# Severe Systemic *Chromobacterium violaceum* Infection: A Case Study of a German Long-Term Resident in French Guyana

**DOI:** 10.3390/tropicalmed9100242

**Published:** 2024-10-15

**Authors:** Caroline Klenk, Miriam Schnieders, Melina Heinemann, Christiane Wiegard, Henning Büttner, Michael Ramharter, Sabine Jordan, Maria Sophia Mackroth

**Affiliations:** 1 I. Medical Department, Division of Tropical Medicine, University Medical Centre Hamburg Eppendorf, Martinistr. 52, 20246 Hamburg, Germany; caroline.klenk@bnitm.de (C.K.); miriam.schnieders@gmx.de (M.S.); michael.ramharter@ctm.bnitm.de (M.R.); s.jordan@uke.de (S.J.); 2 Department of Tropical Medicine, Bernhard Nocht Institute for Tropical Medicine, Hamburg, Bernhard-Nocht-Strasse 74, 20359 Hamburg, Germany; melina.heinemann@usz.ch; 3 I. Medical Department, Division of Infectious Diseases, University Medical Centre Hamburg Eppendorf, Martinistr. 52, 20246 Hamburg, Germany; 4 I. Medical Department, Ultrasound Unit, University Medical Centre Hamburg Eppendorf, Martinistr. 52, 20246 Hamburg, Germany; c.wiegard@uke.de; 5 Department of Medical Microbiology, University Medical Centre Hamburg Eppendorf, Martinistr. 52, 20246 Hamburg, Germany; h.buettner@uke.de; 6 German Centre for Infection Research (DZIF), Partner Site Hamburg-Luebeck-Borstel, Inhoffenstr. 7, 38124 Braunschweig, Germany

**Keywords:** *Chromobacterium violaceum*, case report, liver abscess

## Abstract

*Chromobacterium violaceum* is a Gram-negative, facultative anaerobe proteobacterium. Its natural habitat is water and soil in tropical and subtropical regions. Human infections are characterized by rapid dissemination that can lead to high fatality rates. Here, we describe the first case of a *C. violaceum* infection reported from Germany. A German national with permanent residence in French Guyana contracted a *C. violaceum* infection presumably while bathing in a barrier lake in Brazil. The patient presented with a high fever and a crusty, erythematous skin lesion at an emergency department in Hamburg, Germany. Ultrasound and a CT scan of the abdomen revealed multiple liver abscesses. *C. violaceum* was detected in blood and from aspirates of the liver abscesses, using traditional culture methods and modern molecular assays. Prolonged treatment with meropenem and ciprofloxacin led to full recovery. Rapid pathogen detection and treatment initiation are of high importance in *C. violaceum* infections as mortality rates are overall declining but have still tended to reach up to 25% in recent years in systemic infections. Due to its broad natural drug resistance, antibiotic treatment is challenging. Increased travel activities may lead to more frequent presentation of patients with environmental pathogens of the tropics such as *C. violaceum*.

## 1. Background

*Chromobacterium violaceum* is a Gram-negative, facultative anaerobic, rod-shaped, motile, oxidase-positive saprophytic bacterium. Its normal habitat is water and soil in tropical and subtropical regions. *C. violaceum* is considered facultative pathogenic as it can lead to severe systemic infections and sepsis, even in otherwise healthy people, if entering the bloodstream through the contamination of damaged skin [1,2,3].

Infections are associated with rapid clinical progression and high mortality, and treatment is complicated by *C. violaceum*’s natural resistance to multiple first-line antimicrobials including most beta-lactam antibiotics [2,4,5]. Due to the wide variability in the clinical presentation of this pathogen, it is not possible to readily differentiate clinically between *C. violaceum* infections and other potential microorganisms based on clinical examination alone [3,6,7]. Rapid microbiological detection is therefore important for appropriate treatment, which usually requires the inclusion of a carbapenem or aminoglycoside (Supplementary Table S1). In addition to widely available culture techniques, modern molecular assays such as 16-S ribosomal ribonucleic acid sequencing are increasingly used and could offer additional detection options. Here, we report the first case of a patient with a systemic *C. violaceum* infection in Germany. Rapid identification and appropriate treatment of the pathogen led to a favorable clinical outcome.

## 2. Case Presentation

A 59-year-old male German national presented to the emergency department of the University Medical Centre Hamburg Eppendorf, Germany, with a 5-day history of fever up to 40 °C, diarrhea, nausea and general weakness. Nineteen days prior to presentation, the patient had returned from French Guyana, where he had been residing since 1990. The patient reported that he had suffered from intermittent fever episodes before returning to Germany. He therefore took an oral antibiotic, most likely a beta-lactam antibiotic, which he had bought in a pharmacy in French Guyana without prior medical consultation. The patient did not remember the exact name of the antibiotic. He had also observed a furuncle-like nodule on his abdomen for 4 weeks to which he had applied a disinfectant. As potential risk factors for infections, he reported contact with fresh water by swimming in Lake Tucurui in the Federal State Parà, Brazil, 2 months prior. Other risk factors included regular contact with cattle and sheep and the consumption of non-pasteurized milk in the past.

**Figure 1:**
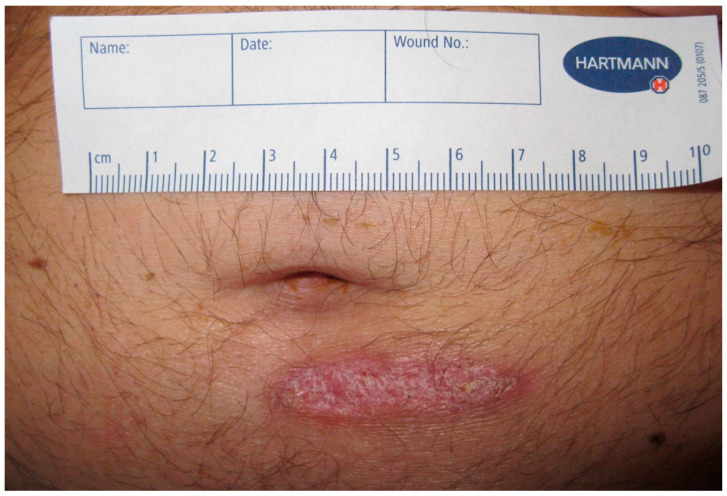
Skin finding on day of admission. Chronic skin lesion on abdomen on day of presentation to emergency room.

There was no relevant past medical history. The patient did not take any regular medication and did not report any known allergies. He worked as a mechanic at a space port in French Guyana and lived with his girlfriend. The clinical examination on admission showed a temperature of 40.3 °C, blood pressure of 146/84 mmHg and a pulse of 112 beats/minute. His respiratory rate was 16 breaths/minute with an oxygen saturation of 94% in room air, and cardiac and pulmonary examinations were unremarkable. The abdominal examination revealed a crusty erythema of 5 cm in diameter below the umbilicus, but no other skin lesions (Figure 1). The full blood count showed leukocytosis of 12.7 × 10^9^ cells/L and hemoglobin of 8.1 mmol/L. Elevated inflammatory markers included C-reactive protein at 1552 nmol/L and procalcitonin at 40.4 µg/L. Liver enzymes and bilirubin were within normal range. LDH was elevated at 5.75 µkat/L. Differential diagnoses at the time of admission included skin abscess and cellulitis of the abdominal wall, gastroenteritis or bacterial infections associated with tropical and subtropical regions including Leptospirosis, Brucellosis, Rickettsiosis or Coxiella infection. Several pairs of blood cultures were taken at admission. A rapid diagnostic test for malaria and thin and thick blood smears were negative. Serologic tests were requested for *Brucella* species, *Leptospira interrogans*, *Rickettsia* (*R.*) *conorii*, *R. typhi*, *R. prowazekii* and *Coxiella burnetii* and returned negative for both IgM and IgG. Microscopic stool examinations for helminth eggs and protozoa were unremarkable. Stool polymerase chain reaction tests for pathogenic bacteria, *Entamoeba histolytica* and *Giardia lamblia,* were negative.

**Figure 2:**
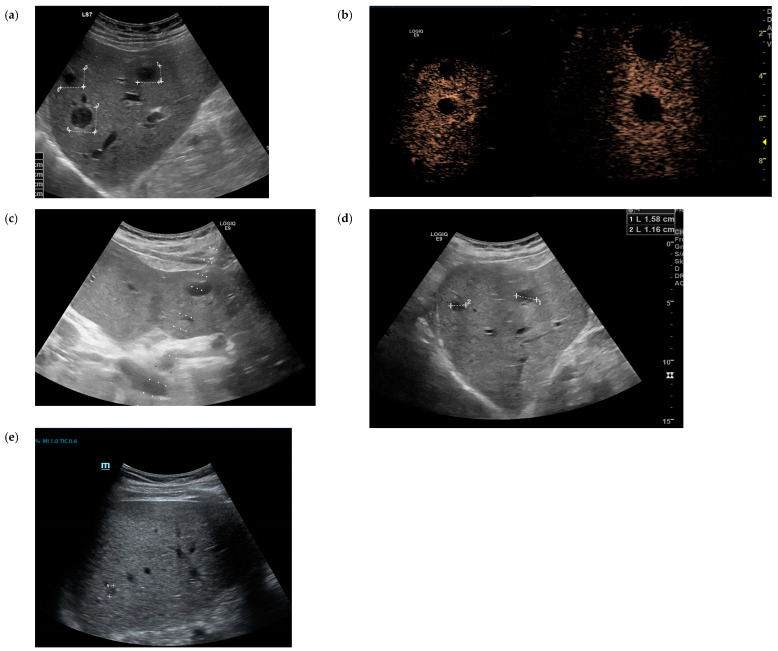
Abdominal and contrast-enhanced ultrasound (CEUS) of the liver. On admission: the liver with multiple small echo-poor lesions sharply delineated from the surrounding tissue (**a**); CEUS: no enhancement of the lesions, but hyperenhancement in the periphery of the abscess (**b**). Ultrasound-guided diagnostic punction of one lesion to gain 3 ml pus (**c**). Progress control after 2 weeks (**d**) and 6 weeks (**e**). The diameter of the multiple echo-poor lesions decreased over 6 weeks under therapy.

The patient was admitted to the infectious disease ward. Empirical antibiotic treatment with parenteral ampicillin/sulbactam 3 g three times daily was initiated with the suspected diagnosis of a soft tissue infection of the abdominal wall. Over the next day, the patient deteriorated clinically with mental confusion and continuing high fever. On the second day after admission, blood culture results were reported to be positive for Gram-negative rods. This led to a change in the empiric treatment to meropenem 1 g three times daily as a differential diagnosis of melioidosis or other unusual Gram-negative pathogens was considered. An abdominal ultrasound revealed multiple echo-poor lesions up to 2 cm in the liver. In a contrast-enhanced ultrasound (CEUS), these lesions showed no enhancement, but strong hyperenhancement of the margin of the lesions in the early contrast phase was observed. This is a typical contrast pattern which is described in pyogenic liver abscesses (Figure 2a,b). Ultrasound-guided diagnostic puncture of one lesion was performed, yielding 3 mL pus (Figure 2c). There was no sonographic sign of an abscess beneath the abdominal skin lesion. CT scans of the thorax, abdomen and brain did not reveal any additional abscesses or infection foci.

Both the liver aspirate and blood cultures were positive for *C. violaceum,* confirmed by bacterial culture on blood agar and chocolate agar where they exhibited a characteristic violet pigment. Additionally, the liver aspirate and blood cultures also confirmed infection with *C. violaceum* by 16-S rRNA sequence analysis using primers 27f and 907r.

The antibiotic treatment was switched to intravenous meropenem and trimethoprim-sulfamethoxazole pending antibiotic sensitivity testing. Sensitivity testing was performed by using the E-test (Epsilometer test) on Mueller Hinton agar. Therefore, a bacterial suspension equivalent to a 0.5 McFarland standard was plated on the agar, and etest strips were placed and incubated at 36 ± 1 °C for 24 h. The results of the E-test are shown in Table 1. The antibiotic treatment was then adjusted to intravenous meropenem (1 g three times daily) and ciprofloxacin (400 mg three times daily) due to estimated susceptibility to these two substances according to PK/PD breakpoints (EUCAST). The patient improved rapidly under the modified antibiotic regimen and was discharged after 12 days of treatment with meropenem/ciprofloxacin. The liver abscesses had already decreased in size (Figure 2d). Markers of inflammation almost normalized to a CRP level of 104 nmol/L and leukocytes of 7.5 × 10^9^ cells/L on the day of discharge. Ciprofloxacin 750 mg two times a day p.o. was recommended as discharge medication for one additional month. The total course of meropenem and ciprofloxacin treatment thus comprised 14 days of meropenem and 6 weeks of ciprofloxacin, of which 14 days were spent hospitalized.

Follow-up examinations took place at regular intervals at the infectious disease outpatient clinic. Blood cultures were taken 3 and 7 weeks after discharge and remained negative. The liver abscesses rapidly decreased in size. Follow-up ultrasound examination 4 weeks after discharge showed only a maximum residual focus of 1 cm in size (Figure 2e). In subsequent examinations, no remaining foci could be detected.

## 3. Discussion

Infections with *C. violaceum* are rare but reported cases have increased in frequency over the last few decades. Varying mortality rates have been published in recent years, ranging from 53 to 80% in systemic infections prior to 2011, but with mortality rates overall declining to approximately 25.4% in recent years due to increased awareness and prompt therapy (Supplementary Table S1). Two studies from Australia reported comparatively low mortality (7 and 0%) and predominantly benign courses with localized infections in a case series of patients with detected *C. violaceum* infections [8,9]. Under-detection and -reporting of benign cases or differences in bacterial virulence or host resistance due to prior colonization by *C. violaceum* may explain this discrepancy. Interestingly, males are predominantly affected, and up to a third of cases involve patients that are immunocompromised or have significant comorbidities [3,10,11,12]. Besides these, specific immunological conditions such as chronic granulomatous disease and glucose-6-phosphate dehydrogenase deficiency in polymorphonuclear leukocytes, both of which are associated with impaired neutrophil function, predispose humans to severe *C. violaceum* infections [3,10,13,14,15].

A PubMed search of case reports involving human infections with *Chromobacterium violaceum* at the time of writing revealed 28 such cases reported over the past five and a half years (from 2019 to the first half of 2024, Supplementary Table S1 [16,17,18,19,20,21,22,23,24,25,26,27,28,29,30,31,32,33,34,35,36,37,38]). Of these 28 cases, 20 patients were male, 5 cases had a fatal outcome and almost all cases were reported from the Northern Hemisphere, albeit originating in or with a travel history to (sub)tropical regions in most instances.

The most common site of entry of the bacterium is through a pre-existing skin lesion that comes into contact with contaminated water or soil. The first symptoms may be nonspecific and include fever, cough, cellulitis or lymphadenitis [1,3,39,40,41]. The infection can subsequently progress rapidly to a systemic infection. At least one-third of patients with *C. violaceum* infections develop localized abscesses in visceral organs, with the liver being the organ that is most commonly involved [3,4,7,16] (Supplementary Table S1). Other manifestations of *C. violaceum* infections include gastrointestinal infections, pulmonary infiltrates or abscesses, meningitis, endocarditis, peritonitis, osteomyelitis, brain abscesses and urinary tract infections [1,3,40,42,43,44,45] (Supplementary Table S1). With these features of multiple abscesses, *C. violaceum* can cause a clinical picture similar to that of other rare environmental pathogens such as *Burkholderia pseudomallei* [17,41].

Infections with *C. violaceum* remain difficult to treat due to its broad natural drug resistance mainly against most beta-lactam antibiotics and due to the rarity of the pathogen itself, especially considering settings outside the tropics. Nearly all reported cases were diagnosed by culturing methods, but diagnosis still remains challenging, primarily due to a lack of awareness [3,5,7,11].

Most published cases were successfully treated with a carbapenem or aminoglycoside combined with trimethoprim-sulfamethoxazole or fluoroquinolones depending on the results of prior susceptibility testing. Parenteral antimicrobial treatment is most often used for 2–4 weeks followed by maintenance therapy with an oral agent such as trimethoprim-sulfamethoxazole or a fluoroquinolone for 1–3 months to prevent relapse [3,11,15,40,41] (Supplementary Table S1).

Our case illustrates a successful outcome following the rapid pathogen detection of *C. violaceum* in blood cultures and the aspirate of liver abscesses. Despite the prolonged duration of infection, multiple liver abscesses and the severe condition at admission, treatment with meropenem and ciprofloxacin led to rapid recovery. This emphasizes the importance of early pathogen detection and appropriate antimicrobial therapy for this difficult-to-treat pathogen.

## 4. Conclusions

Heightened awareness of rarer pathogens like *C. violaceum,* considering increased travel and exposure to soil and freshwater in tropical regions, is essential for achieving a successful treatment outcome in returning travelers presenting with a plausible clinical picture, particularly when common causative pathogens have been ruled out. Therefore, it is important to consider *C. violaceum* infection as a potential differential diagnosis in skin and soft tissue infections and prolonged fever, especially in patients with remote abscesses and relevant travel or exposure history. Prompt microbiological evaluation is crucial for accurate diagnosis and appropriate management. In addition, physicians should advise patients with skin injuries to avoid swimming in freshwater bodies, particularly in tropical regions, to reduce the risk of infections with aquatic pathogens such as *C. violaceum*.

## Figures and Tables

**Table 1:** *C. violaceum* susceptibility results of Epsilometer test.

Antibiotic	Minimal Inhibitory Concentration
piperacillin	32 µg/mL
ceftazidim	≥256 µg/mL
meropenem	1 µg/mL *
ciprofloxacin	0.016 µg/mL *

* According to the EUCAST PK/PD table, susceptibility was estimated for meropenem and ciprofloxacin.

## Data Availability

The original contributions presented in the study are included in the article/Supplementary Material, further inquiries can be directed to the corresponding author.

## Supplementary Materials

The following supporting information can be downloaded at: https://www.mdpi.com/article/10.3390/tropicalmed9100242/s1, Table S1: Case reports of human infections with *Chromobacterium violaceum* in 2019–2024. References [16,17,18,19,20,21,22,23,24,25,26,27,28,29,30,31,32,33,34,35,36,37,38] are cited in the supplementary materials.
