# Supplementary material for: Severe Systemic Chromobacterium violaceum Infection: A Case Study of a German Long-Term Resident in French Guyana
Source: Trop Med Infect Dis. 2024 Oct 15;9(10):242. doi: 10.3390/tropicalmed9100242 (PMC11511043; doi:10.3390/tropicalmed9100242)
Supplement: Supplementary file 1 [file tropicalmed-09-00242-s001.zip › tropicalmed-3225712-supplementary.pdf]

Case Reports of human infections with *Chromobacterium violaceum* 2019-2024

Reference	Age	Sex	Comorbidities	Country	Exposure	Presentation	Diagnosis	Treatment	Outcome
Li, Kun et al. 2024 [16]	62	m	diabetes	China	possibly soil/water contact	septic shock, liver abscess, lower limb infection	NGS, culture (blood)	imipenem + cilastatin, levofloxacin + meropenem	good
Wang, Xueqing et al. 2024 [4]	15	m	none	China	possibly previous accident	sepsis, purpura fulminans, liver abscess	culture (blood, sputum)	meropenem + vancomycin, co-trimoxazole + amikacin + doxycycline	good
Nam, Bùi Văn et al. 2024 [17]	13	f	pulm. TB, ITP	Vietnam	unknonw	skin abscess, cellulitis	culture (wound)	cefepime + amikacin, linezolid + meropenem, cefepime + vancomycin, ciprofloxacin	good
	8	f	pulm. TB	Vietnam	unknown	sepsis, osteomyelitis	culture (blood)	cephalosporin, cefoperazone + tobramycin, ceftazidime + linezolid, ciprofloxacin + meropenem, levofloxacin + co-trimoxazole	good
Yavuz, Lemis et al. 2023 [18]	17	f	none	UAE	possibly home swimming pool	necrotic cellulitis	culture (wound)	amoxicillin/clavulanic acid, clindamycin, cefepime, ciprofloxacin	good
Barnes, Gonzales et al. 2023 [19]	2 m	m	CDG	USA	playing outside after hurricane	skin lesions, septicemia	culture (wound, tissue and blood)	vancomycin + ceftriaxone, meropenem + levofloxacin, voriconazole + co-trimoxazole	good

Gomez, Sanz et al. 2023 [20]	12 y	f	none	Argentina	wounded by thorn in a lagoon	abscesses ankle, bilateral infiltrates	culture (wound, tracheal), Maldi-TOF	vancomycin + clindamycin + ceftriaxone, imipenem + amikacin, ciprofloxacin + co-trimoxazole	good
Attonito, Tomasello et al. 2023 [21]	27 y	f	unknown	USA	unknown, Marine recruit	skin and liver abscesses	culture (blood and wound)	meropenem + ciprofloxacin	good
Anjaneyan, Bhaskaran et al. 2022 [22]	6 m	m	suspected CGD and TB	India	unknown	skin lesions and pulmonary infiltrates	culture (blood and wound)	ceftriaxone + aciclovir, meropenem + vancomycin, ciprofloxacin + amikacin	Fatal
Mohammed, Sajana et al. 2021 [23]	48 d	m	none	India	suspected due to contaminated water during baptism	septic shock, liver abscess	culture (blood)	meropenem + teicoplanin, ciprofloxacin + co-trimoxazole	good
Laghu, Yanagawa et al. 2021 [24]	48 y	m	spinal cord injury	Nepal	suspected inlying catheter	UTI	culture (urine)	ofloxacin	good
Zhang, Li et al. 2021 [25]	50 y	m	unknown	China	leg puncture during farm work	skin lesion leg	culture (wound)	piperacillin/tazobactam, levofloxacin	good
Chowdhurry, Lee et al. 2021 [26]	30 y	m	discoid lupus	Azores/UK	hot springs during vacation in the Azores	skin lesions, pulmonary abscesses	culture (wound), Maldi-TOF	amoxicillin/clavulanic acid, piperacillin/tazobactam + clindamycin, meropenem + ciprofloxacin + gentaymicin	good
Cubides Diaz, Arsanios Martin et al. 2021 [27]	47 y	m	polycystic kidney	Cololmbia	unknown, employee in hotel sector	periareolar soft tissue infection	culture (wound)	clindamycin, doxycycline, tigecycline	good

Lang, Wang et al. 2021 [28]	15 y	m	possible immune deficiency, previous *C. violaceum* infection	China	working in a field	several abscesses	NGS, negative culture (blood, wound)	amikacin + meropenem	good
Er, Chun et al. 2021 [29]	11 y	m	none	Malaysia	fell into wet field	open radius and ulnar fracture, liver abscess	culture (wound)	cefuroxime, ciprofloxacin, cefepime + amikacin	good
Takeda, Tanaka et al. 2021 [30]	49 y	m	none	Japan	fell into muddy rice field	multiple closed fractures	culture (blood)	cefotiam	fatal
Sachu, Antony et al. 2020 [31]	76 y	f	multimorbid	India	suspected due to washing clothes in lake	several skin lesions	culture (blood and wound)	amoxicillin/clavulanic acid + clindamycin, meropenem	fatal
Mazumder, Sadique et al. 2020 [32]	40 y	m	none	Bangladesh	accident in muddy field	skin lesions,	culture (blood and wound), 16S rRNA gene sequencing	cefixime, meropenem + ciprofloxacin	good
Moretti, Baggi Menozzi et al. 2020 [33]	83 y	f	multimorbid	Switzerland	hurt during gardening, swimming in pool	skin lesion leg	culture (blood)	amoxicillin/clavulanic acid, piperacillin/tazobactam + ciprofloxacin + co-trimoxazole	good
Thwe, Ortiz et al. 2020 [15]	37 y	m	G6PDH deficiency, previous *C. violaceum* infection	USA	unknown, suspected relapse	septicemia	culture (blood), 16S rRNA gene sequencing	meropenem + doxycycline	good

Sharmin, Jahan et al. 2019 [34]	40 y	f	hypertension	Bangladesh	unknown	septicemia, pneumonia	culture (tracheal)	meropenem + moxifloxacin, ceftriaxone + co-trimoxazole	fatal
Olalekan, Itua et al. 2019 [35]	31 y	m	suspected chronic illness	Nigeria	unknown, water from bore holes	pleural empyema	culture (pleural empyema)	amoxicillin/clavulanic acid + ceftriaxone, ciprofloxacin	good
	2 m	m	none	Nigeria	unknown, water from bore holes	UTI	culture (urine)	cefotaxime	good
	5 y	m	unknown	Nigeria	unknown, water from bore holes	UTI	culture (urine)	ceftriaxone	good
Jedruszczak, Wegrzyn-Bak et at. 2019 [36]	55 y	m	multimorbid	Czech Republic	unknown	sepsis	culture (blood)	ciprofloxacin, levofloxacin + meropenem	good
Bansie, Harkisoen et al. 2019 [37]	37 y	m	CDG	Suriname	visit to creek recreational area	septicemia, liver abscesses	culture (wound, blood, faecal)	amikacin + clindamycin + flucloxacillin, meropenem + ciprofloxacillin	good
Dzupova and Benes 2019 [38]	54 y	m	unknown	Czech Republic	diving holiday in Thailand	ear infection, septic shock	unknown	unknown	fatal

Supplementary Table S1, Source: PubMed search, ‚Chromobacterium violaceum‘ + case reports + 2019 -2024
